# The 40-year history of modeling active dendrites in cerebellar Purkinje cells: emergence of the first single cell “community model”

**DOI:** 10.3389/fncom.2015.00129

**Published:** 2015-10-20

**Authors:** James M. Bower

**Affiliations:** Numedeon, Inc.Ashland, OR, USA

**Keywords:** Purkinje cells, modeling, cerebellum, cerebellar, dendrite, active conductnaces, history

## Abstract

The subject of the effects of the active properties of the Purkinje cell dendrite on neuronal function has been an active subject of study for more than 40 years. Somewhat unusually, some of these investigations, from the outset have involved an interacting combination of experimental and model-based techniques. This article recounts that 40-year history, and the view of the functional significance of the active properties of the Purkinje cell dendrite that has emerged. It specifically considers the emergence from these efforts of what is arguably the first single cell “community” model in neuroscience. The article also considers the implications of the development of this model for future studies of the complex properties of neuronal dendrites.

## Introduction

Analysis of the complex behavior of the mammalian cerebellar Purkinje cell has contributed significantly to our understanding of the role and function of active electrical properties in central nervous system dendrites. Further, as reviewed in this article, the study of the active properties of the dendrites of this neuron is unusual for neuroscience in the extent to which it has involved an interaction between “realistic” biophysically accurate computer models and laboratory-based experiments. Accordingly, in addition to considering the possible functional significance of the active dendritic properties of the mammalian Purkinje cell, this article also recounts in some detail the evolution of the models on which that analysis is based. Ideally, this history should serve as a model for the analysis of all aspects of the functional organization of nervous systems.

It turns out that the co-dependence between modeling and experimental studies of Purkinje cells was established at the earliest stages of study of this neuron’s complex electrical behavior. This early interaction between models and experiments was induced by a claim made by Llinas et al. (1968) based on experimental results, that Purkinje cell dendrites were electrically active. That claim, based on experimentally obtained time delays in shock induced field potentials recorded at different depths of the alligator cerebellum, was immediately challenged by Calvin and Hellerstein (1969) who, citing Rall’s (1964) pioneering cable modeling results, suggested that such delays were likely a simple consequence of passive dendritic current conduction alone. In defending their interpretation, Llinas and colleagues asserted in return that models based on volume conductors rather than cable models were a more appropriate basis for the analysis of extracellular field potentials. A few months later, Zucker (1969) entered the debate by actually performing calculations comparing both types of models, concluding that neither approach, in its classical form, could resolve the issue. However, Zucker pointed out that similarities in simulated field potential results recently obtained from the more active cable theory models for mitral cells developed by Rall and Shepherd (1968) likely supported Llinas’ original interpretation. In response, Calvin suggested that Zucker’s model had too many free parameters, and defended his own argument as based on “*the simplest possible model consistent with our objective* *(to demonstrate that a) commonplace explanation for conduction velocities was as good as the more esoteric*” (Calvin, 1969, p. 637). It took 10 more years and the development of experimental brain slice procedures and the application of intracellular recording techniques for Llinas and Sugimori (1980a) to provide conclusive experimental evidence that Purkinje cell dendrites are in fact electrically active.

**Figure 1:**
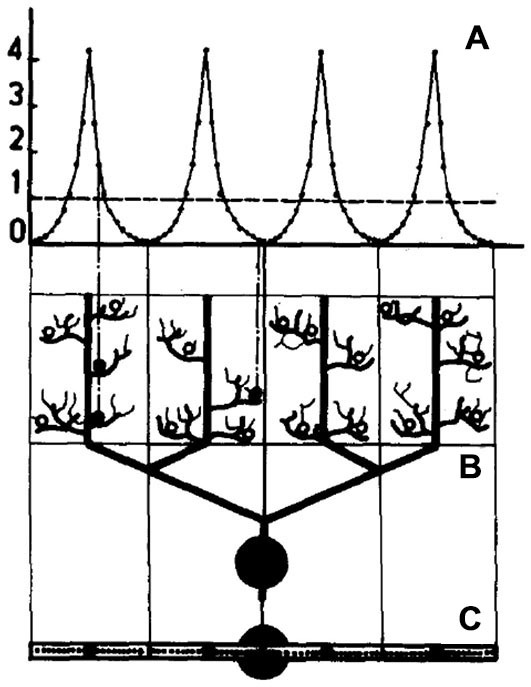
**Schematic representation of a model Purkinje cell model simulated in Pellionisz and Szentágothai (1974).** The dendritic tree is divided into four non-overlapping synaptic territories meant to represent the main Purkinje cell dendritic branches. **(A)** shows the distribution of parallel fiber synapses on each dendritic branch, **(B)** is the modeled Purkinje cell viewed in a parasagittal plane and **(C)** is the Purkinje cell viewed from the top. The fine structure within each branch in this figure is only for illustrative purposes and did not influence the summation of synaptic inputs. Reproduced with permission from Pellionisz and Szentágothai (1974).

It is important to point out that while references to computational modeling was at the heart of this very early controversy, no effort was actually made by any of the discussants to actually build a model of the Purkinje cell dendrite (Calvin and Hellerstein, 1969). Instead, the first model of a Purkinje cell dendrite was published by Pellionisz and Szentagothai as the last of a series of early cerebellar network modeling studies (Pellionisz, 1970; Pellionisz and Szentágothai, 1973, 1974). As shown in Figure 1, in that model, the complex Purkinje cell dendrite was represented by only four branches in which synaptic influences were calculated independently, using a simple algebraic summation. On reaching threshold, each branch independently generated dendritic spikes which were then simply summed at the soma. Comparing results of network simulations using these four branch Purkinje cells to previous results with no dendritic structure these authors concluded that: “*the simulation experiments are giving quite strong hints in favor of the importance of dendritic geometry*” (Pellionisz and Szentágothai, 1974, p. 28).

**Figure 2:**
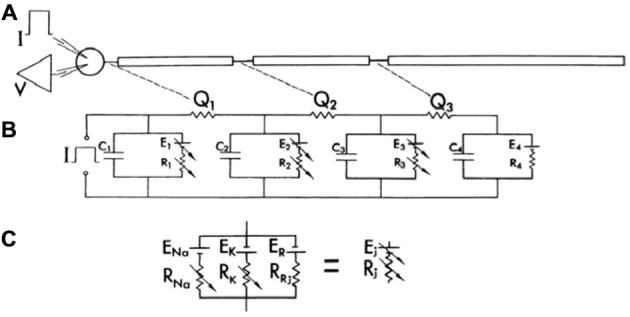
**The first published compartmental model of a Purkinje cell, consisting of a soma and three dendritic compartments.** As shown in **(A)**, the model consisted of a soma and three dendritic compartments, with only the soma and the first two dendritic compartments receiving synaptic input. **(B)** Represents the electrical diagram representing the model. Compartments are separated by a resistance Qi. Potential across the soma and the first two dendritic compartments is represented by a variable battery (Ej) and a variable resistor (Rj) to simulate synaptic input in parallel with the membrane capacitance (Cj). The last compartment (4), had a constant resting emf. **(C)** Further describes the electrical variable battery and resistance. Further explanation for the structure of the model can be obtained from the original manuscript. The model was used in conjunction with experimental data to support the hypothesis that the climbing fiber made multiple synaptic inputs on the proximal Purkinje cell dendrite. Reproduced with permission from Llinas and Nicholson (1976).

Perhaps reflecting the influence of the original debate between Llinas and Calvin and Hallerstein in the 1960’s, Llinas and Nicholson (1976) published the first true compartmental model of the Purkinje cell dendrite to specifically test new speculations on cerebellar physiology based on field potential recordings. In this case, the experiments involved climbing fiber-evoked responses in cat cerebellar cortex. As shown in Figure 2, while their compartmental model included conductances represented with Hodgkin Huxley model parameters (Hodgkin and Huxley, 1952), the model included only three dendritic compartments whose active properties were limited to the synapses.

**Figure 3:**
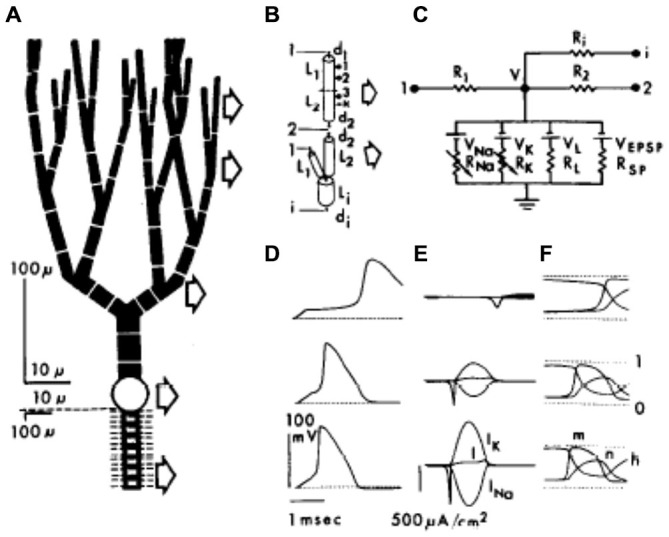
**The first full compartmental model of the Purkinje cell dendritic tree represented by 62 dendritic compartments (A), with each of the compartments (B) simulating ionic conductances using an equivalent electrical circuit (C). (D–F)** show the responses of three different compartments after a simulated somatic current injection (dendritic branch point, upper row; soma middle row; node of Ranvier, lower row). Reproduced with permission from Pellionisz and Llinás (1977).

One year later, as shown in Figure 3, Llinas now working with Pellionisz, published the first compartmental Purkinje cell model with more a more complex dendritic tree (Pellionisz and Llinás, 1977). Using as a base a previously published compartmental model of a spinal motorneuron (Dodge and Cooley, 1973), the new Purkinje cell model consisted of 62 compartments with the soma and initial segment incorporating Hodgkin Huxley channels (Hodgkin and Huxley, 1952). With this model the authors sought, for the first time, to use the model to replicate actual experimental responses of frog Purkinje cells including: (1) the rapid “antidromic” decrement in action potential amplitude in the dendrite following somatic current injection (Llinas et al., 1969b; Freeman and Nicholson, 1975); (2) the orthodromic activation of Purkinje cells following parallel fiber stimulation (Eccles et al., 1966a); and (3) the spike burst resulting from climbing fiber synaptic input (Eccles et al., 1966b, 1967). While the authors’ state explicitly in their article that compartmental modeling is an essential technique to: *“(handle) a partially or totally active dendritic tree*” (Pellionisz and Llinás, 1977, pg. 37) the model they reported still included no active voltage dependent dendritic conductances.

## Deducing Function from Structure

In their original justification for building the first more realistic Purkinje cell model, Llinas and Pellionisz explicitly state that: “*Rigorous mathematical models of the electrical activity of central neurons (are) a powerful tool to test and interpret experimental data*” (Pellionisz and Llinás, 1977, p. 37). However, the model they actually published was clearly built to demonstrate the plausibility of dendritic mechanisms the authors had previously inferred from physiological results. In reviewing the cerebellar (and generally the neuroscience) modeling literature, this type of “demonstration model” is still the most common, with most published models specifically built to demonstrate the plausibility of one prior interpretation or another. Accordingly, these models are not intent on testing or interpreting experimental data, but instead on demonstrating the plausibility of a particular idea.

As described in the rest of this article, models can, and in the case of the cerebellar Purkinje cell have, instead been used to reveal unexpected and new interpretations of experiment and function. These models however, have been built first and foremost on anatomical structure and to replicate basic physiological responses, making as few functional assumptions as possible. As also demonstrated in the following history, models of this sort are also more likely to result in the kind of model sharing by multiple investigators in multiple laboratories which in principle can lead to cooperation, accelerating progress and understanding.

**Figure 4:**
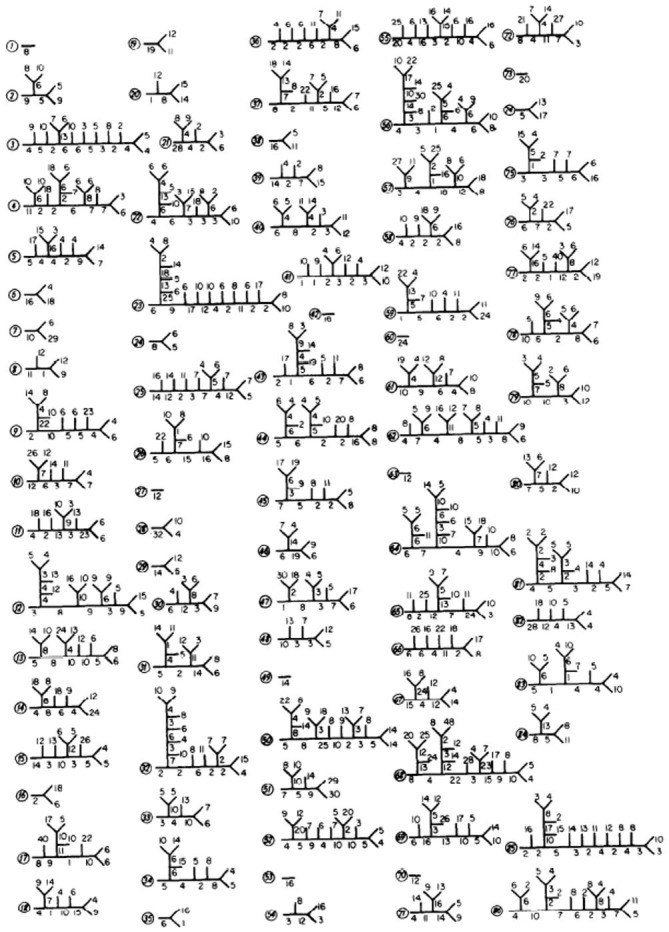
**From Shelton (1985) showing details of each of the modeled Purkinje spiny dendritic branches.** Used with permission from Shelton (1985).

The first published Purkinje cell model that explicitly set out to deduce function from structure, without assuming the function to begin with was published by Shelton (1985) using, for the first time, an actual anatomical dendritic reconstruction of a real Purkinje cell (Figure 4). While structurally realistic, this model, like the earlier Purkinje cell models, did not include active dendritic properties, an omission justified by the authors assertion that: “*the part of the dendritic tree of the Purkinje cell which is thought to be essentially passive forms a very large fraction of the total membrane surface area of the cell*” (Shelton, 1985, p. 111), although the author later notes that dendritic passivity is an assumption of the model, rather than a conclusion. Instead the model was used to provide a description of the expected passive electrical properties of the Purkinje cell given the morphology of its dendrite. This was accomplished by tuning the model to replicate experimentally observed differences in dendritic and somatic input conductances. It should be noted that while this model was built on an actual anatomical reconstruction of a rat Purkinje cell, for technical reasons the only physiological data available was from Guinea Pigs. Accordingly the author “stretched” the rat dendrite to better resemble a Guinea Pig Purkinje cell. In regard to the possible active properties of the Purkinje cell dendrite, Shelton’s explicitly stated that his exploration of the passive properties of the dendrite should “*form the substrate for extensions which would treat more complex properties*” (Shelton, 1985, p. 111).

Reflecting Shelton’s original intent, the next realistic model of the cerebellar Purkinje cell was published by Rapp et al. (1994, p. 114) explicitly as “*an essential step—a skeleton—for constructing biologically more realistic models of PC dendrites*”. These authors, who based their model on Guinea Pig morphology, also explicitly tested Shelton’s speculated on the possible influence of active synaptic conductances on passive membrane properties by applying the first synaptic inputs to the dendrite (Rapp et al., 1992). The Rapp et al. modeling publications also, for the first time, included new experimental data obtained by the author’s specifically to parameterize the model, while also considered in some detail the application of newly developed parameter estimation methods for large compartmental models (Holmes and Rall, 1992). Rapp et al. (1994) also tested their results using different reconstructed dendritic morphologies. Harkening back to the original controversy about the appropriate form of modeling to explore dendritic function, these authors also explicitly compared compartmental modeling results to analytical cable model solutions pioneered by Rall (1964), Calvin and Hellerstein (1969), Zucker (1969) and Segev et al. (1985). In publishing their model, Rapp et al. (1994, p. 114) however, explicitly stated, once again, that it was now essential that Purkinje cell models, “*incorporate a variety of non-linear voltage- and ligand-gated channels that we know exist in the Purkinje cell dendrite*”.

Returning to the community model sub-theme for this article, in addition to being the first Purkinje cell model (and one of the first in neuroscience) to be based on an actual anatomically reconstructed dendrite, the Shelton model was also the first Purkinje cell model whose components were reused by other modelers (Bush and Sejnowski, 1991; Genet et al., 2010; Blum and Wang, 1990; Brown et al., 2011), in each case adding active dendritic properties to the model. However, once again, in each modeling study, the intent was to demonstrate a previous idea about the functional significance of this property.

**Figure 5:**
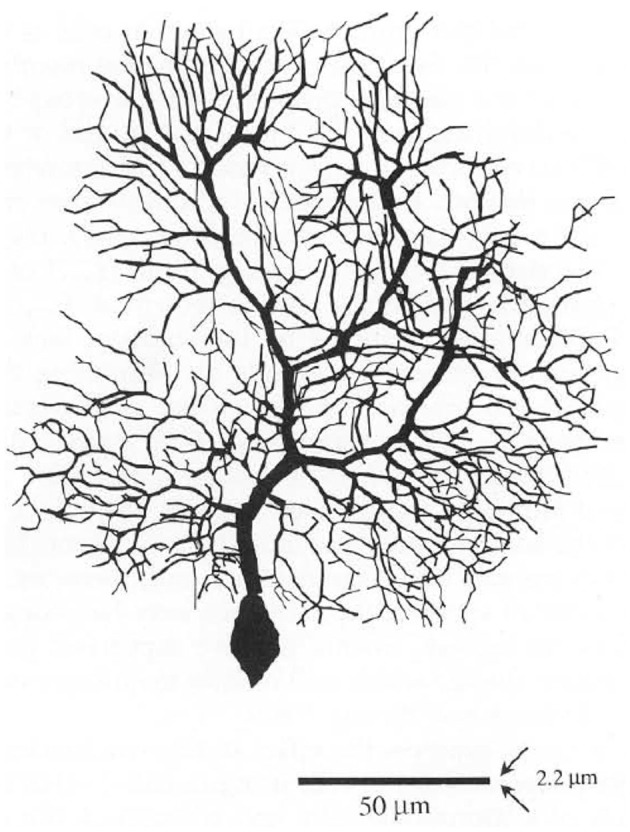
**The original Rapp et al Purkinje cell model, reconstructed from a Guinea Pig.** Reproduced with permission from Rapp et al. (1992).

While Shelton’s model was the first realistic Purkinje cell model, and was used by others to build new models, these versions of the Shelton models have not generated further versions. Likely this is due in part to the fact that these models were intended to demonstrate, rather than discover function, but also because the models were not written in a form easily transmitted to others. Instead, it is the original Rapp et al. (1992, 1994) Purkinje cell model (Figure 5) that lead to the model that has emerged as “*among the most successful, cited, and re-used/updated in computational neuroscience*” (Ascoli, 2007, p. 156). It is clear from the history that my laboratory played a critical role, first by translating the Rapp model into GENESIS, the general purpose simulator also built in my laboratory (Bower and Beeman, 1995, 2007) and second, because by adding a full set of active conductances to the model, independent of a set of underlying functional assumptions or objectives. This second feature of our modeling efforts I think is especially important, because it means that other investigators don’t have to “buy” our interpretations or assumptions about function.

**Figure 6:**
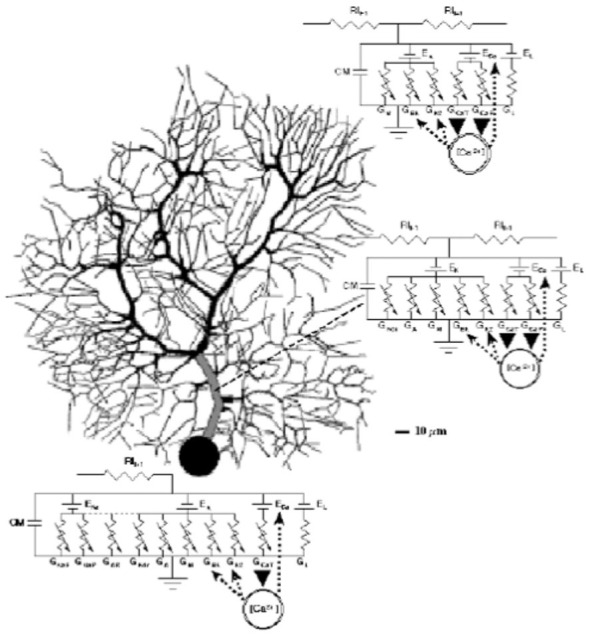
**Schematic description of the De Schutter and Bower Purkinje cell model with equivalent circuit diagrams for the modeled ionic conductance included in each section of the cell.** Reproduced with permission from De Schutter (1999).

After obtaining a copy of the model from Rapp and colleagues even before their final article was published (De Schutter et al., 1993; Jaeger et al., 1993), we used GENESIS to included 10 active conductances differentially distributed in the soma and dendrite, parametrized on data from a wide range of *in vitro* voltage clamp experiments. The initial model-based results of the consequences of active dendritic processes for the basic physiological responses of recorded Purkinje cells were published in a series of three articles published in De Schutter and Bower (1994a,b,c). The first of these articles De Schutter and Bower (1994a) explicitly extended the work of Shelton (1985) and Rapp et al. (1992, 1994) with an analysis of the electrical structure of the Purkinje cell dendrite now including active voltage dependent conductances (Figure 6). The second article De Schutter and Bower (1994b) explored dendritic responses to climbing fiber input extending the study of the model to understand the possible influence of background excitatory synaptic inputs again first explored by Rapp et al. (1992, 1994) but now also including inhibitory synapses. The third article considered for the first time the response of an active Purkinje cell dendrite to the type of synaptic activity expected to result from stimulus driven input (De Schutter and Bower, 1994c). As the first neuronal model to use concurrent supercomputers (De Schutter and Bower, 1992), these simulations involved a much more extensive test of parameter space than previously possible, demonstrating that modeled responses were quite robust to changes in its primary parameters. Importantly for the reuse of this model by others, the use of the GENESIS simulation system specifically developed for sharing realistic neurobiological models (Bower and Beeman, 1995) made the Purkinje cell model one of the first if not the first published online (De Schutter, 1994). Again, availability of the model to anyone—its construction within a modeling platform, and I believe its focus on physiological rather than functional interpretations has led this model to be one of the first, if not the first community model in neuroscience.

## Emergence of a Community Purkinje Cell Model

The articles by Rapp et al. (1992, 1994) and De Schutter and Bower (1994a,b,c) have collectively been cited more than 500 times, with the first description of the active Purkinje cell model De Schutter and Bower (1994a) responsible for almost half those citations. Importantly, the model, we now refer to as the “R-DB model”, has formed the basis for considerable subsequent work from my own students both within my laboratory (Jaeger et al., 1996; Baldi et al., 1998; Sultan and Bower, 1998; Jaeger and Bower, 1999; Mocanu et al., 2000; Santamaria et al., 2002, 2007; Santamaria and Bower, 2004; Lu et al., 2005, 2009; Cornelis et al., 2010) and within their own independent laboratories and research (Staub et al., 1994; De Schutter, 1998; Vos et al., 1999; Howell et al., 2000; Steuber and De Schutter, 2001, 2002; Gauck and Jaeger, 2003; Solinas et al., 2003, 2006; Kreiner and Jaeger, 2004; Koekkoek et al., 2005; Santamaria et al., 2006, 2011; Shin and De Schutter, 2006; Shin et al., 2007; Steuber et al., 2007; Achard and De Schutter, 2008; De Schutter and Steuber, 2009; Anwar et al., 2012, 2013, 2014; Coop et al., 2010; Tahon et al., 2011; Cao et al., 2012; Couto et al., 2015). Perhaps more importantly the R-DB model has become a true “community model” as it is now being used by a growing number of authors as a base for further modeling work outside its laboratories of origin (Coop and Reeke, 2001; Mandelblat et al., 2001; Miyasho et al., 2001; Roth and Häusser, 2001; Chono et al., 2003; Khaliq et al., 2003; Steuber and Willshaw, 2004; Ogasawara et al., 2007; Yamazaki and Tanaka, 2007; Kulagina et al., 2008; Traub et al., 2008; Brown et al., 2011; Brown and Loew, 2012; Forrest et al., 2012; Forrest, 2015; Masoli et al., 2015). Several of these modeling efforts have now started their own lineage sequences, with, for example, the adaptation of the original R-DB Model by Miyasho et al. (2001), being further extended by Chono et al. (2003), Kulagina et al. (2008), and Brown et al. (2011). Importantly, the model has also been translated from the original GENESIS files to multiple other modeling platforms. As described in this next section, much of that modeling work has been focused on replicating and understanding the complex responses of Purkinje cells resulting from the active properties of its dendrite.

One of the first uses of the R-DB Model outside of my own laboratory’s lineage, explicitly tested the model’s ability to replicate PC responses obtained from new *in vitro* experimental studies using ion channel blockers (Miyasho et al., 2001). Using dendritic morphology from the rat (Shelton, 1985) parameterized with data from the R-DB Model, Miyasho et al. (2001) modified channel descriptions and conductance densities to reproduce the repetitive Ca^2+^ spike firing they had found experimentally after the application of TTX *in vitro*. Importantly, these authors also refined the kinetics of the K^+^ delayed rectifier current, applying a new mechanism for calculating intracellular Ca^2+^ concentration while also changing the Ca^2+^ sensitivity of the calcium-activated dendritic K^+^ conductance. With these changes, the model was extended to replicate physiological responses including: (1) characteristic Ca^2+^ dendritic spikes in the presence of TTX; (2) repetitive Ca^2+^ spiking patterns resulting from the presence of TTX; (3) the lack of Ca^2+^ spikes found after application of a P-type Ca^2+^ channel blocker; (4) the slow onset of the Ca^2+^ spikes in response to a depolorizing current steps; and (5) the marked shortening of the Ca^2+^ spike onset seen in the presence of 4-AP. Two years later, Chono et al. (2003) further refined the Miyasho et al. (2001) model by adding new channel descriptions as well as refinements in the conductance values for the simulated Ca^2+^ and Ca^2+^ dependent K^+^ channels. These enhancements have since been incorporated into Purkinje cell modeling efforts by other groups (Traub et al., 2008; Brown et al., 2011).

Having extended the ability of the R-DB Model to replicate physiological data obtained under new pharmacological conditions, Miyasho et al. (2001) then explored the possible contribution to dendritic calcium spike generation of two low threshold dendritic calcium related conductances they had recently discovered in their own experimental studies (Watanabe et al., 1998). Adding Ni^2+^ sensitive Ca^2+^ channels to the dendrites, these authors demonstrated that the model could now replicate the longer onset Ca^2+^ spikes found in the presence of Ni^2+^.

**Figure 7:**
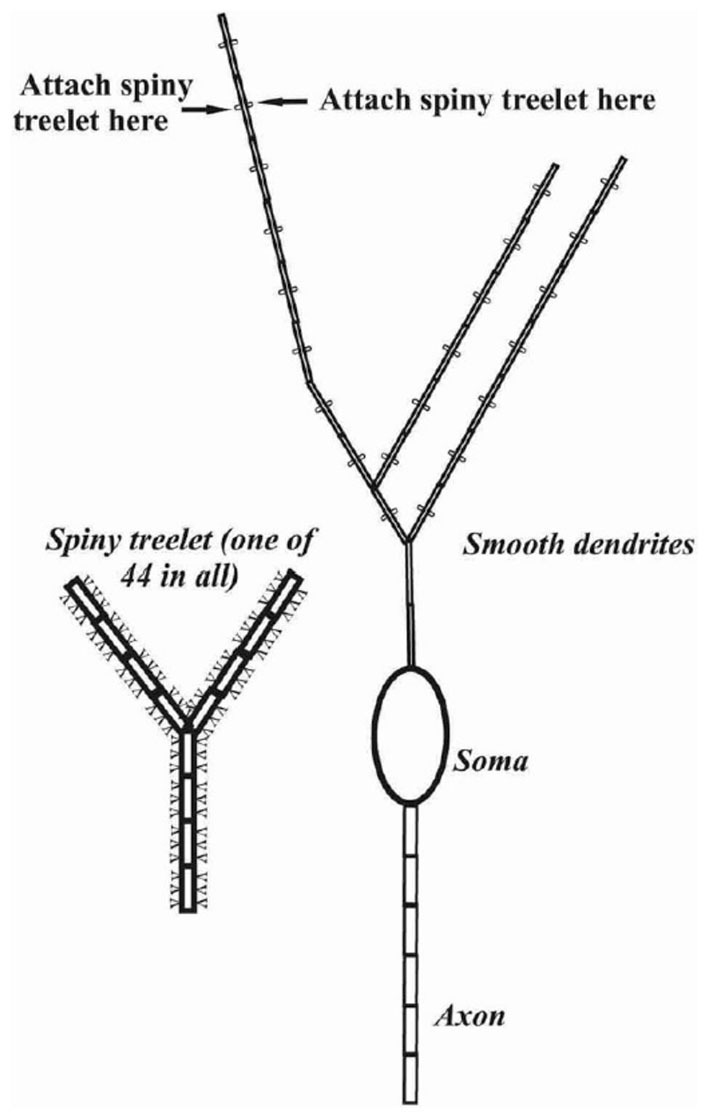
**Schematic representation of the cerebellar Purkinje cell model in Traub et al. (2008).** Reflecting the focus of the study on putative gap junctions between the initial axon segments of Purkinje cells, this axonal region was represented by six compartments while the dendrite was reduced to 553 compartments with a particular emphasis on the spiny branchlets. Used with permission from Traub et al. (2008).

This is the kind of cumulative refinement and advancement that can best, or perhaps can only take place with community models. However, equally important to changes in the structure of a community model, is the use of that model to explore new forms of behavior or perform new forms of analysis not considered by the original model’s authors. To this end, several authors have used the R-DB Model in a reduced from to more closely examine neuronal dynamics (Mandelblat et al., 2001; Fernandez et al., 2007). In a series of publications, Brown et al. (2011) adapted the original R-DB Model to explore how mechanisms at the subcellular (biochemical) levels could be linked to somatic output (Rapp et al., 1992; Brown et al., 2011; Brown and Loew, 2012). While building a new model in Fortran, Traub et al. (2008) never-the-less extended R-DB Model parameters to explore the possible role of gap junctions between the initial axon segments of Purkinje cells in cerebellar cortical oscillations. To do so, he reduced overall dendritic complexity while maintaining a “realistic” path from the distal dendrite to the soma (see Figure 7).

The R-DB Model is also now being used in the context of both subcellular and network level scales. Sub-cellularly, the model has been used as a base to consider the effects of molecular or biophysical mechanisms on Purkinje cell function (Holmes and Rall, 1992; Brown et al., 2011; Brown and Loew, 2012), and to provide a larger context for studies of subcellular modeling of calcium diffusion (Santamaria et al., 2006, 2011; Anwar et al., 2012) as well as biophysical mechanisms of synaptic plasticity (Vladimirescu et al., 1981; Antunes and De Schutter, 2012; De Schutter, 2013). The model has also been used to build network level simulations in reduced (Yuen et al., 1995; Coop and Reeke, 2001; Sarro, 2004), and full form (Howell et al., 2000; Solinas et al., 2003; Santamaria et al., 2007).

The R-DB model has also been applied to new analytical studies, including, for example, questions involving the information processing potential of dendrites (Coop et al., 2010) as well as possible spike coding strategies (Jaeger and Bower, 1999; Steuber and De Schutter, 2001, 2002; De Schutter and Steuber, 2009). Efforts have also been made to link the structure of the R-DB Model to the kind of analysis involved in the field of artificial neural networks (Steuber and De Schutter, 2001; Sarro, 2004).

Finally, the R-DB Model is being used as a base for assessing modeling technology itself, including parameter estimation techniques (Van Geit et al., 2007) and the relationship between parameter variations and modeling results (Achard and De Schutter, 2008).

## Understanding Purkinje Cell Responses to Different Types of Input

Having established the community status of the R-DB model, the remainder of this article will consider what has been learned as a result of the use of the model. While general reuse and improvement are important, ultimately the utility of any model, whether used by the community or not, is its ability to generate and truly test hypothesis regarding function (De Schutter, 1999). This is also the most complex and challenging application for any model, especially given the tendency of all scientists to want to see what they want to see. Accordingly especially important, in my view, is a clear establishment of community standards for model performance. In this regard, the next section is organized around a set of Purkinje cell behaviors actually identified by Pellionisz and Llinás, (1977, p. 42) as necessary for, “*any Purkinje cell model which claims to be adequate*”. As described in subsequent sections of this article, all of these behaviors turn out to depend on the active properties of the Purkinje cell dendrite, and replicating and understanding these core response properties has provided the basis for further analysis of the functional significance of active dendritic processes.

### Antidromic Spike Activation of the Purkinje Cell Dendrite

**Figure 8:**
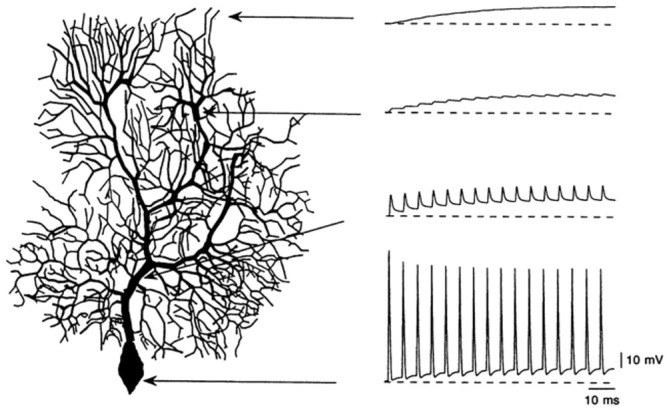
**Simulation of the lack of antidromic action potential dendritic invasion in a modeled Purkinje cell following simulated current injection in the soma.** Used with permission from Rapp et al. (1994).

Perhaps the most straightforward characteristic Purkinje cell response, identified by Pellionisz and Llinas, is the fact that action potentials generated in the Purkinje cell soma do not propagate into its dendrite (Figure 8). At the time of the first Purkinje cell modeling studies, this lack of antidromic dendritic invasion had already been predicted based on field potential recordings (Llinas et al., 1969b; Freeman and Nicholson, 1975), although the phenomenon was not directly observed experimentally until much later (Llinas and Sugimori, 1980b). In the early passive models, the lack of back propagation was attributed to the relative surface area of the cell dendrite compared to its soma (Pellionisz and Llinás, 1977; Rapp et al., 1994). This explanation was further elaborated in another passive modeling study using parameters obtained from the R-DB Model (although with different dendritic morphology) as due to a large cumulative impedance mismatch resulting from the high branching density of the Purkinje cell dendrite (Roth and Häusser, 2001). With respect to active dendritic mechanisms the models have shown that the very low Na^+^ channel density in Purkinje cell dendrites provides no mechanism to overcome these morphological effects (De Schutter, 1999; Kitamura and Häusser, 2011) a result also reported in models of other types of mammalian neurons (Vetter et al., 2001).

### Responses to Somatic Current Injection

**Figure 9:**
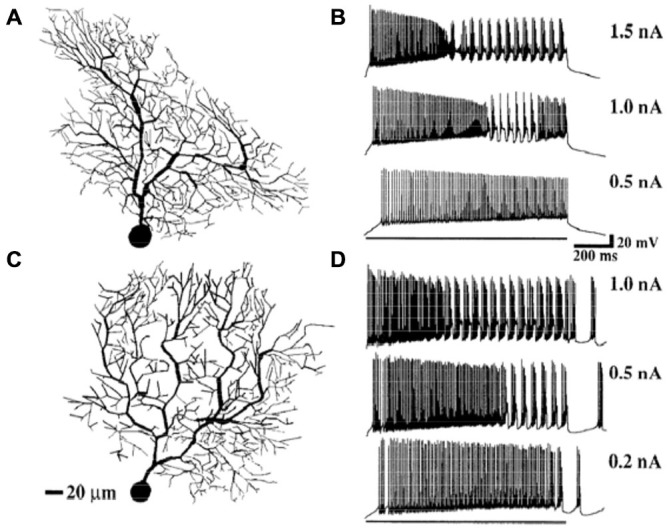
**Simulation of somatic responses to three different amplitude synaptic current injections in two models with different dendritic morphologies.** Model **(A)** produced responses **(C)**, Model **(B)**, responses **(D)**. The results specifically replicate the typical rapid spiking to bursting pattern seen *in vivo* in response to somatic current injection. Given that identical amounts of current are injected, and each model has the same electrical parameters, the variations in response properties are due to the different morphologies of the modeled cells. Reproduced with permission from De Schutter and Bower (1994a).

It has been known since intracellular recordings were first made in Purkinje cells, that their response to current injection is complex (Llinas and Sugimori, 1980b). The modeling results shown in Figure 8 were obtained from a passive Purkinje cell dendritic model after current injection in the soma. In fact, as shown in Figure 9, current injection in a real Purkinje cell (and the active R-DB model), produces a much more complex pattern of somatic and dendritic activity (Gähwiler and Llano, 1989; Hirano and Hagiwara, 1989; Kaneda et al., 1990; Regan, 1991; Wang et al., 1991; Lev-Ram et al., 1992). In part for this reason, although not explicitly a part of the original Pellionisz and Llinás (1977) standard for Purkinje cell models, the ability to replicate the results of *in vitro* current injection studies has become the defacto standard for testing and tuning active Purkinje cell models (Bush and Sejnowski, 1991; De Schutter and Bower, 1994b; Coop and Reeke, 2001; Mandelblat et al., 2001; Miyasho et al., 2001; Forrest et al., 2012).

While a full description of the mechanisms responsible for these *in vitro* response patterns is beyond the scope of this article, the general result from modeling studies is that this behavior of the Purkinje cell is a function of a complex interaction between all its biophysical and anatomical properties (De Schutter, 1999). This conclusion is somewhat in contrast with the more typical analysis from experimental studies which usually associate different features of the *in vitro* response properties to specific kinds of afferent input (Gähwiler and Llano, 1989; Hirano and Hagiwara, 1989; Kaneda et al., 1990; Regan, 1991; Wang et al., 1991; Lev-Ram et al., 1992; Miyasho et al., 2001), i.e., fast events associated with somatic action potential generation; the somewhat slower Ca^2+^ related dendritic bursting behavior assumed to be related to climbing fiber inputs; and longer time course events assumed to be influenced by granule cell related synaptic inputs (Traub et al., 2008; Brown et al., 2011; Isope et al., 2012; Kitamura and Kano, 2012). The models clearly show that these responses are actually related to the entire structure of the Purkinje cell and the interaction between its different afferent inputs. Bursting responses to climbing fiber inputs, for example, are also dependent on the level of background granule cell synaptic input.

**Figure 10:**
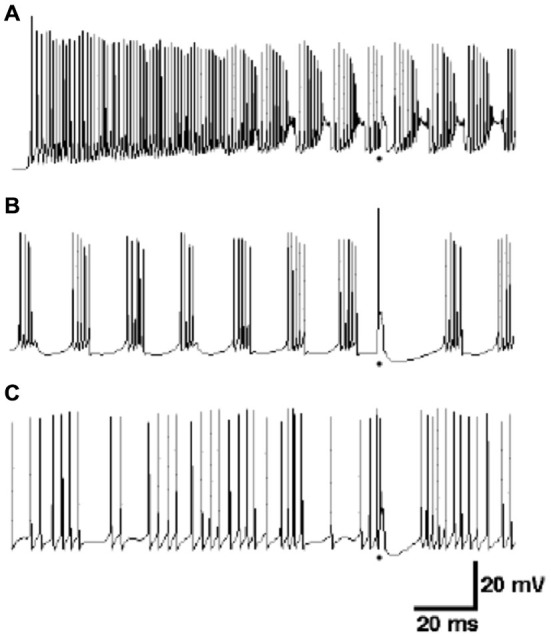
**Comparison of responses of the R-DB Model in the absence of background synaptic input to the dendrite (A), in the presence of only excitatory synaptic input (B) and both excitatory and inhibitory input (C).** As described in the text, the firing pattern in **(A)** resembles Purkinje cell activity recorded *in vitro*, while **(C)** resembles *in vivo* activity. Figure used with permission from De Schutter (1999).

It turns out that this co-dependence discovered in the models sheds new light on the importance of the experimental conditions under which Purkinje cells are studied. For example, it has actually been known for many years that the spontaneous behavior of Purkinje cells *in vitro* is quite different from the spontaneous behavior of Purkinje cell *in vivo* (Llinas and Sugimori, 1980b). As shown in the modeling results of Figure 10A, *in vitro* behavior consists of relatively rapid (usually >100 Hz) action potentials, interrupted periodically by spontaneous dendritic calcium spikes. In contrast, as simulated in Figure 10C, Purkinje cells *in vivo* generate spontaneous action potentials at lower frequencies (usually <80 Hz) that are quite irregular. Dendritic Ca^2+^ spikes are also believed to only appear *in vivo* in response to climbing fiber inputs (Llinas and Nicholson, 1976) whereas *in vitro* they occur spontaneously. Understanding how the response properties of the cell changes *in vitro* is important given how much of the study of the active properties of neurons has been done using this technique. What modeling results have suggested is that it is the lack of background synaptic input in what is essentially a deafferented brain slice preparation that is reasonable for differences in *in vivo* and *in vitro* behavior (Jaeger et al., 1996). Perhaps particularly important in Purkinje cells which are known to receive 150,000 excitatory parallel fiber inputs. However, when provided with background excitatory input alone, the R-DB Model produced a pattern of output that resembled neither the *in vitro* nor *in vivo* conditions (Figure 10B; De Schutter, 1999). Instead, replication of *in vivo* patterns required spontaneous input from both excitatory and inhibitory synaptic inputs (Figure 10C). Accordingly, the models predict both in single cell (Jaeger et al., 1996; Watanabe et al., 1998) and network form (Howell et al., 2000) that normal Purkinje cell behavior likely depends on current from constant background synaptic inputs, interacting with the active Ca^2+^ and K^+^ dependent channels in the dendrite and soma (De Schutter, 1998). Experimental studies specifically designed to test these modeling predictions are consistent with this interpretation (Jaeger and Bower, 1999; Kreiner and Jaeger, 2004). Realistic models have therefore provided an essential tool to relate *in vitro* response properties to the natural *in vivo* behavior of Purkinje cells especially challenging given the complexity of this cells active dendritic properties.

### Purkinje Cell Responses to Climbing Fiber Activation

**Figure 11:**
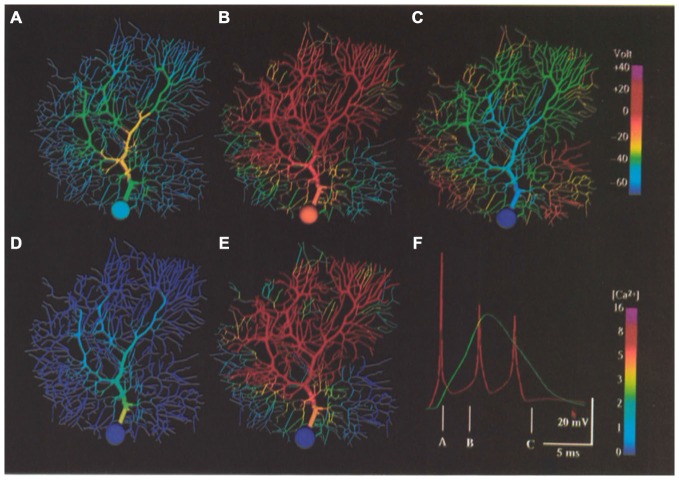
**False color representation of membrane potential and Ca^2+^ concentration during simulation of a climbing fiber input. (A)** Membrane potential 1.4 ms after beginning of the resulting complex spike. **(B)** Membrane potential 4.0 ms after beginning of complex spike. **(C)** Membrane potential 10.0 ms after beginning of a complex spike (after the last somatic action potential). **(D,E)** Submembrane Ca’+ concentration at same times as **(A,B)**, respectively. **(F)** Complex spike as it appears in the soma (red) and distal dendrite (green) at the same times represented by **(A–C)** as indicated. Note the non-linear [Ca’+] scales. Figure used with permission from De Schutter and Bower (1994a).

The fact that the Purkinje cell responds to climbing fiber activation *in vivo* with a burst of action potentials has been known for many years (Eccles et al., 1966b). In fact as already noted, the first compartmental Purkinje cell model was specifically constructed to test this experimentally derived prediction (Llinas and Hillman, 1969) that this response behavior was a consequence of the multiple synaptic contacts distributed over the Purkinje cell dendrite by a single climbing fiber (Llinas and Nicholson, 1976), with subsequent modeling focused on the actual biophysical mechanisms responsible for producing the “oscillatory wavelets” or “spike burst” characteristic (see Figure 11F) of climbing fiber responses (Pellionisz and Llinás, 1977). At the time, these authors concluded that the different peaks in the somatic burst response were generated by repetitive firing of the initial segment of the axon rather than by an active dendritic mechanism as had been previously proposed (Eccles et al., 1966b).

Neither Shelton (1985) nor Rapp et al. (1992, 1994) attempted to replicate Purkinje cell responses to climbing fiber activation, however, this was an important component of the initial analysis of the active dendritic and somatic model of De Schutter and Bower (1994b). In fact, after tuning model parameters to replicate responses to somatic current injection data, the ability of the model to generate climbing fiber burst responses without further tuning parameters was the first indication of the model’s likely realism (see Figure 11). As already described, the model predicted that the correct *in vivo* form of the climbing fiber response was dependent on background patterns of excitatory and inhibitory synaptic input. However, analysis of the model also predicted that the dendritic response was dependent on the activation of P type Ca^2+^ channels in both the cells smooth and the spiny dendrites, with the duration of the dendrite spike being regulated by Ca^2+^ activated K^+^ conductances. The modeling results also suggested that the biphasic reversal potential of the climbing fiber induced EPSP, with an early portion reversing before the later portion (i.e., the climbing fibers “duel reversal potential”) previously shown experimentally (Llinas and Hillman, 1969) and attributed solely to the spatial distribution of climbing fiber synapses (Llinas and Nicholson, 1976) was also likely dependent on the details of the active properties of the Purkinje cell dendrite. Further, an unexpected but important prediction of the model was that climbing fiber activation resulted in substantial increases in intracellular calcium not only in the smooth dendrites, where climbing fiber synapses actually terminate, but also in the smallest spiny dendritic branches receiving granule cell synaptic inputs (Gundappa-Sulur et al., 1999; Lu et al., 2009) again showing the interrelatedness of the anatomical and physiological components of the dendrite. The involvement of the entire dendrite in the climbing fiber event was simultaneously shown experimentally (Konnerth et al., 1992; Miyakawa et al., 1992). The model also predicted that inhomogeneity in local levels of calcium activation in the dendrite did not depend on a non-uniform distribution of Ca^2+^ channels as had previously been proposed Tank et al. (1988) and Llinas and Sugimori (1992). Instead the pattern of calcium response was a consequence of the non-uniform geometry of the Purkinje cell dendrite, and likely varied from Purkinje cell to Purkinje cell. Thus, unlike Rapp et al. (1994), who reported little effect of individual dendritic variations on cellular passive properties, the active model suggested that differences in individual Purkinje cell morphologies might, in fact have important functional significance.

### Replication of the Simple Spike Firing of Purkinje Cells

The final, and it turns out most difficult standard for Purkinje cell modeling proposed by Pellionisz and Llinás (1977) was the ability to replicate simple spike firing in response to granule cell (parallel fiber) input. This is, of course, mor difficult because, in principle, understanding the important features of this behavior is likely linked directly to questions of neuronal coding, which we really know about nothing about. Never-the-less, it is the attempt to replicate this behavior of the Purkinje cell with the R-DB Model has produced the most interesting and provocative structural and functional predictions resulting in several new hypotheses regarding the cell’s overall function and in fact the function of the cerebellum itself (Bower, 2002). The following sections will consider several examples.

#### The Natural Function of the Purkinje Cell Dendrite Depends on the Presence of Background Synaptic Inputs

**Figure 12:**
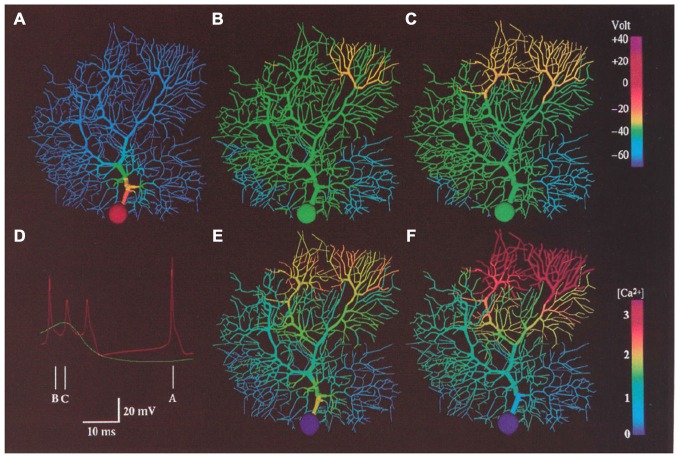
**False color representation of membrane potential and Ca^2+^ concentration during a 2.0 nA current injection in the soma of the modeled Purkinje cell.** Simulated membrane potential is shown during a somatic action potential **(A)**, at the beginning of a dendritic spike **(B)** and 1.6 ms later **(C)**. **(D)** shows predicted somatic (red) and dendritic (Green) membrane potential at the times indicated. **(E,F)** indicated submembrane Ca^2+^ concentration at the same time as **(B,C)** respectively. Reproduced with permission from De Schutter and Bower (1994b).

As already described, one important prediction of the R-DB Model is that the natural behavior of the Purkinje cell dendrite depends on the presence of continuous excitatory and inhibitory synaptic input from the granule cell pathway. Again, while background excitatory granule cell (parallel fiber) synaptic activity had been anticipated for some time to influence ongoing Purkinje cell firing (Llinas et al., 1969a), in order to get realistic patterns of spiking out of the active Purkinje cell model it was necessary to also add background inhibitory synaptic inputs (De Schutter and Bower, 1994a). These modeling efforts resulted in several predictions. First the model predicted that Purkinje cell behavior was dependent on the ability of the soma, itself, to spontaneously generate action potentials. This ability has now been demonstrated experimentally (Pugh and Raman, 2009), and has recently also been further studied using a model derived from the R-DB line (Forrest et al., 2012). Second, as shown in Figure 12, the model predicted that the large intrinsic voltage gated currents and not the relatively smaller currents associated with synaptic activation most influenced ongoing somatic spiking (Jaeger et al., 1996; De Schutter, 1998; Jaeger and Bower, 1999). In fact, the model predicted that the Purkinje cell dendrite is actually dominantly a current sink rather than a source, making the behavior of the Purkinje cell very different from that of a traditional integrate and fire neuron (see below). Further, the model suggested that background spontaneous parallel fiber inputs had much less of an effect on the actual timing of Purkinje cell spikes than did inhibitory synaptic input (Jaeger et al., 1996). While a full description of the dendritic dynamics underlying this behavior is beyond the scope of this chapter (for more details, see De Schutter and Bower, 1994b,c; Jaeger et al., 1996; De Schutter, 1999; Jaeger and Bower, 1999), experimental (Jaeger and Bower, 1999; Womack and Khodakhah, 2002a,b, 2004; Womack et al., 2004; Santamaria et al., 2007) and subsequent R-DB Model related studies (Howell et al., 2000; Miyasho et al., 2001; Coop et al., 2010; Brown et al., 2011; Forrest et al., 2012) have supported these unexpected but model-predicted interactions between the Purkinje cell dendrite and soma.

#### “Dendritic Democracy” and the Influence of Distal Synaptic Inputs

The influence of excitatory synaptic input in such a large dendrite has been a central issue for Purkinje cell modeling for many years. In fact, the publication by Llinas et al. (1968) that sparked the first consideration of modeling in Purkinje cells (Calvin and Hellerstein, 1969; Calvin, 1969; Zucker, 1969) started by posing the following fundamental question: “*In studying the anatomy of the Purkinje cell, one wonders how the distal region of (these large) dendrites can act upon the soma and axon …”* (Llinas et al., 1968, p. 1132). That article went on to identify two possibilities: *(i) by direct electrotonic spread from the distal dendrite to the soma, or (ii) by the initiation of action potentials or local responses which can be conducted either in an all- or-none manner or in a decremental fashion down to the axon.”* (Llinas et al., 1968, p. 1132). Considering this question was also a primary objective of the modeling efforts of both Shelton (1985) and Rapp et al. (1992, 1994); who both predicted, based on their passive models, that the Purkinje cell dendrite was actually electrotonically compact and therefore that distal synaptic inputs, in principle should have an influence on the soma similar to those more proximal. Shelton specifically describes the functional significance of the high passive dendritic input resistance as “*a specialization which optimizes the dendrites for signaling (the soma) with minimum (synaptic) attenuation”* (Shelton, 1985, p. 127). This apparent characteristic of the passive electrical properties of the Purkinje cell dendrite has been described as promoting “dendritic democracy” so that: “*somatic EPSP amplitude is only weakly dependent on synaptic location on Purkinje cell spiny branchlets*” (Roth and Häusser, 2001, p. 469).

In the description of the behavior of their passive models, Llinas et al. (1968), Pellionisz and Llinás (1977), Shelton (1985) and Rapp et al. (1994) all mentioned that this baseline “dendritic democracy” likely only applied to the passive electrical properties of the dendrite, and was therefore likely to change with the addition of active conductances. Shelton (1985, p. 128), specifically predicted that the addition of synaptic conductances would likely “*swamp*” the passive membrane conductivity significantly extending the electrotonic length of the dendrite. Actual simulations by Rapp et al. (1992, 1994) again using a passive model, supported Shelton’s speculation, predicting that individual parallel fiber synapses “*essentially loose their functional meaning (in the presence of large amounts of background synaptic input) and only activation of a large number of parallel fibers will significantly displace the membrane potential*” (Rapp et al., 1992, p. 530).

**Figure 13:**
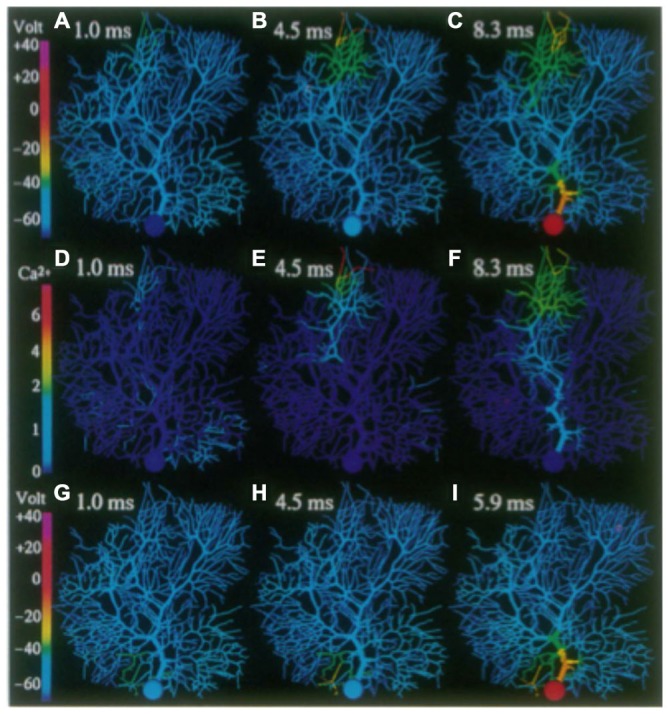
**False color images of the response of the R-DB Model to a synchronous synaptic input on a distal (A–F) and proximal (G-I) branchlet.** Membrane potential in **(A–C)** and **(G–I)**. **(D–F)** Submembrane Ca^2+^ concentrations corresponding to activity in **(A–C)**. Reproduced with permission from De Schutter and Bower (1994c).

In fact, in our active dendritic models adding both synaptic conductances as well as the large voltage dependent dendritic Ca^2+^ related membrane conductances did further extend the electrotonic length of the dendrite (De Schutter and Bower, 1994b) a modeling result subsequently confirmed experimentally (Staub et al., 1994; Ascoli, 2007). However, as described in the third article in the series (De Schutter and Bower, 1994c), the addition of dendritic voltage dependent Ca^2+^ membrane conductances revealed a new and unexpected biophysical mechanism in which synchronously activated granule cell inputs induced a sub-threshold Ca^2+^ dependent amplification mechanism that restored “democracy” to the dendrite even in the presence of ongoing background synaptic input (Figure 13). While Llinas had suggested the general possibility that active membrane properties could facilitate the influence of synapses on the soma, and Shelton (1985, p. 128), specifically speculated that “*active dendritic spikes or active graded potentials may act as a booster mechanism to overcome the electrotonic lengthening of the dendrite due to synaptic activation*”, the specific mechanism that emerged from the R-DB Model was unexpected. Instead of being dependent on a dendritic calcium spiking mechanism as previously assumed (Pellionisz and Szentágothai, 1974), the mechanism involved activation of a sub-spiking threshold calcium event (Figure 13). As a result, in these simulations, a small number of synchronously activated granule cell synaptic inputs produced a similar level of depolarization in the soma regardless of where they were located on the dendrite, a form of “dendritic democracy” that turned out to be dependent and reflect the actual temporal pattern of synaptic input. Further, and importantly, while generating a somatic spike in the passive dendritic models required the activation of large numbers of excitatory synapses (Llinas and Sugimori, 1980b; Rapp et al., 1992, 1994), the active model predicted that somatic spike generation due to synchronously activated synaptic input required an order of magnitude fewer active synapses (De Schutter and Bower, 1994c). This prediction was subsequently confirmed experimentally (Isope and Barbour, 2002). The model has also predicted a similar amplification effect on synchronized inhibitory inputs (Solinas et al., 2006).

#### Purkinje Cells are Tuned to Operate in Context of Activity in the Overall Cerebellar Cortical Network

Another very general but critically important insight gained from the models is that understanding neuronal function requires that a neurons physiological properties be considered in the context of the network in which they are embedded, and in particular in the context of the temporal and spatial patterns of afferent information converging on that cell as a consequence of network structure. While this might at first seem completely obvious, by embedding the R-DB Model within realistic network simulations, very specific new predictions were obtained on this relationship (Santamaria et al., 2007). As with single cell modeling, it is our view that for models to generate new predictions (rather than simply demonstrate pre-conceived functional notions) network level modeling must also be tested against a clearly defined set of physiological behaviors, preferably not yet well understood (Bower, 1990). To be able to interpret the significance of the active properties of the Purkinje cell dendrite with respect to network organization, it will be necessary to first consider these network level physiological behaviors.

**Figure 14:**
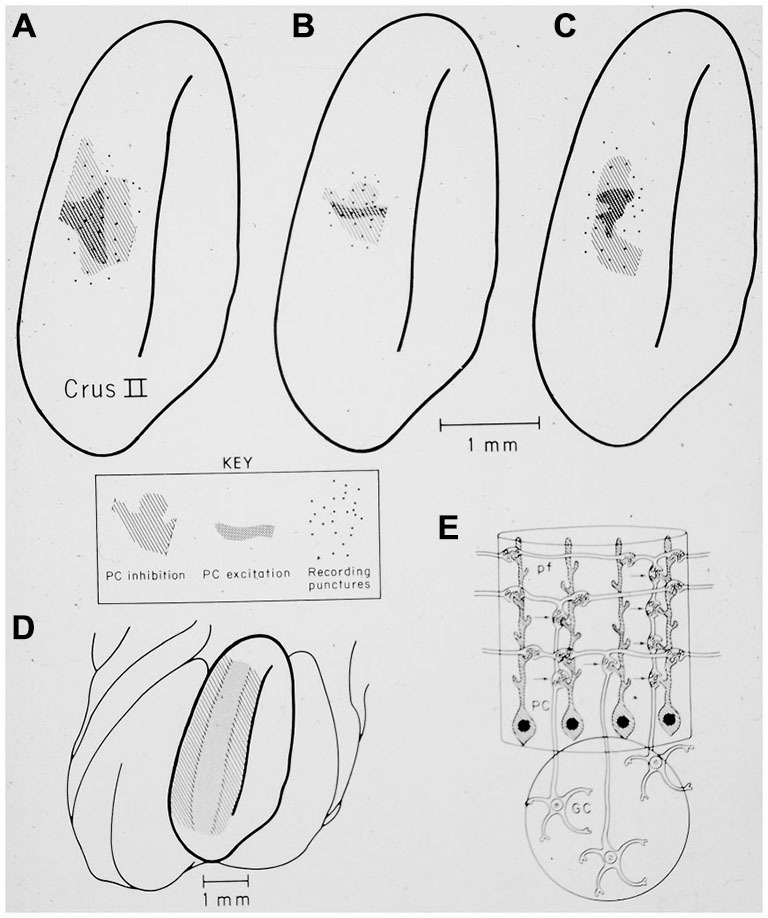
**(A–C)** show the restricted spatial pattern of excitatory (dark stippling) and inhibitory (light hatching) Purkinje cell responses following peripheral stimulation in three experiments. The stimulus activated only granule cells beneath the region of excitatory PC responses. **(D)** shows the expected pattern of activation if parallel fibers drove Purkinje cell responses. **(E)** Original drawing from Llinas (1982) illustrating the hypothesis that synapses associated with the ascending segment of the granule cell axon drove the excitatory Purkinje cell responses. Reprinted with permission from Bower and Woolston (1983).

As it turns out the original motivation for cerebellar modeling in my laboratory was to investigate an unexpected and counterintuitive pattern of Purkinje cell responses to peripheral sensory stimuli (see Figure 14) observed *in vivo* (Bower and Woolston, 1983). Specifically, the spatial extent of Purkinje cell responses to peripheral stimuli was found to be far more restricted than was expected from the organization of cerebellar cortical circuitry and in particular the considerable anatomical spread of the parallel fibers within cerebellar cortex (Eccles et al., 1967, 1971; Bell and Grimm, 1969; Bower and Woolston, 1983). Results consistent or directly supporting this finding have now been reported in numerous subsequent experiments (Kolb et al., 1997; Cohen and Yarom, 1998; Lu et al., 2005; Holtzman et al., 2006; Heck et al., 2007; Rokni et al., 2008; de Solages et al., 2008; Brown and Ariel, 2009; Walter et al., 2009; Dizon and Khodakhah, 2011).

In the original experimental studies published in the early 1980’s, the restricted extent of Purkinje cells activated by peripheral stimuli was interpreted in the most obvious way by suggesting that parallel fibers were simply less influential on Purkinje cell output than had previously been assumed (Bower et al., 1980; Bower and Woolston, 1983). However, it was not clear why responding Purkinje cells were only found over regions of active granule cell layer. In Llinas (1982) suggested that this experimental result (Bower et al., 1980; Bower and Woolston, 1983) could be explained if Purkinje cells were driven by synchronous input from synapses made by granule cells as they ascend through the molecular layer past the Purkinje cell dendrite (Mugnaini, 1972), but not by more asynchronous parallel fiber inputs (Llinas, 1982). Llinas, however, attributed this effect simply to the reduced synchrony of parallel fiber inputs.

When considered now in the context of the R-DB Modeling results, this explanation seemed perfectly consistent with the relative lack of direct influence of background parallel inputs on Purkinje cell spiking, combined with the amplification mechanism for synchronize excitatory inputs (De Schutter and Bower, 1994c). Accordingly it was fully expected that the R-DB Model, once placed in a network context, would confirm Llinas speculation, that the effect simply had to do with the timing of the different synaptic inputs. It was surprising therefore, that even the most desyncronized pattern of parallel fibers, still induced the dendritic boosting mechanism driving somatic output (Santamaria et al., 2007). Resolving this difference between experimental data and modeling results required the introduction of feed-forward inhibitory synaptic inputs to the network model (Santamaria et al., 2007; Walter et al., 2009).

The modeling efforts intended to replicate the restricted pattern of Purkinje cell activation to afferent input (Bower and Woolston, 1983), have perhaps most fundamentally changed how we think of cerebellar cortical processing (Bower, 2010). While most previous theories of cerebellar function have focused on the parallel fiber system as the primary driver of Purkinje cell somatic firing (Braitenberg, 1967; Marr, 1969; Albus, 1971; Pellionisz and Szentágothai, 1974; Medina and Mauk, 2000; Vetter et al., 2001; Heck and Sultan, 2002; Ito, 2006; Kitamura and Kano, 2012), model analysis suggests that it is actually the synapses associated with the ascending segment of the granule cell axon, firing nearly synchronously and not the parallel fibers, which influence spike timing in the soma (De Schutter and Bower, 1994c). Further, the model has also predicted that ongoing somatic spiking activity is not directly influenced by synaptic input, but instead is mediated through the large active conductances in the soma and dendrite. In this view, the synchronous ascending input simply modifies the timing of action potentials that would have been generated anyway (Santamaria and Bower, 2004).

**Figure 15:**
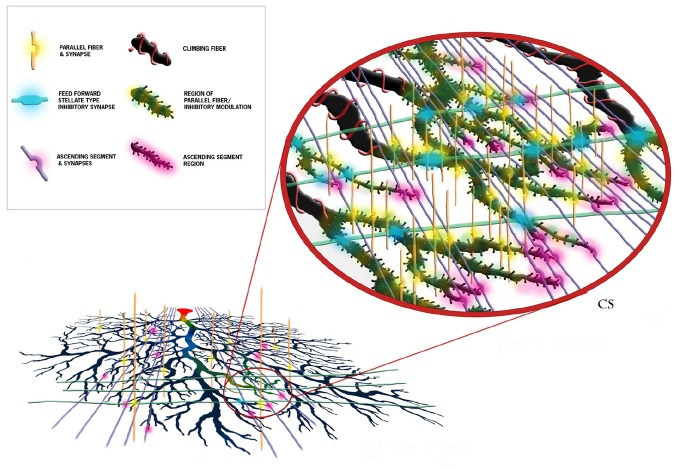
**Schematic representation of the proposed synaptic and functional structure of cerebellar Purkinje cells.** Each element and region is color coded as shown in the figure legend. The diagram demonstrates that the influence of ascending segment synapses must traverse regions of the spiny dendrite influenced by parallel fibers and molecular layer inhibitory interneurons. This is predicted to form the anatomical basis for parallel fiber modulation. Reproduced with permission Bower and Bower (2013).

As just briefly described, perhaps one of the more important consequences of the modeling effort has been to clarify and make quite clear predictions regarding different functional roles of the parallel fibers and the ascending segment synapses of the same granule cell axon (Bower, 1997c). While parallel fiber inputs modulates the overall state of the dendrite, it is the ascending segment inputs that more closely drive output. Interestingly, this functional difference turns out to actually be manifest in the fine physical structure of the Purkinje cell dendrite itself. As shown in Figure 15, anatomical studies have demonstrated that the synapses associated with the ascending granule cell axon segments are found only on the distal regions of the dendrite (Gundappa-Sulur et al., 1999; Lu et al., 2009), where our network models predict that these synapses will be synchronously active in response to afferent mossy fiber stimuli (Santamaria et al., 2007). Our single cell models predict that the active properties of the dendrite mediate a boosting mechanism allowing this distant input to influence somatic spiking (De Schutter and Bower, 1994c). Anatomical studies have also shown that parallel fiber synapses are found primarily on the more proximal spiny dendrites (Gundappa-Sulur et al., 1999; Lu et al., 2009), where both the network (Santamaria et al., 2007) and single cell (Jaeger et al., 1996) models suggest they interact with feed forward inhibition to regulate the activation state of the large dendritic voltage dependent Ca^2+^ and Ca^2+^ activated K^+^ conductances. This places parallel fibers in a position to influence or modulate, the response of the dendrite to the synchronous ascending segment synapses. The models predict that this modulation by the parallel fibers and molecular layer inhibition is mediated through their control of the membrane voltage in the dendrite, and thus the state of activation of the large dendritic voltage dependent conductances. Thus the same active voltage dependent dendritic conductances are responsible for mediating the amplification mechanism for distal synchronous ascending segment inputs as well as the spiking behavior of the soma in general (Bower, 2010). As an aside, these results also suggest that climbing fiber activation resets these modulatory mechanisms (Bower, 1997b), a role consistent with another original prediction of the R-DB Model, that calcium influx from climbing fiber activation would spread to the distal most regions of the dendrite (De Schutter and Bower, 1994b). In this way, the use of anatomically and physiologically realistic models has resulted in predictions that, in effect, merge the anatomical and physiological properties of this cell. In my view, this is what is meant by exploring structure function relationships. Importantly again, the models were not built with these relationships in mind, they came out of running the models.

### Implications and the Importance of Community Models

In summary, while it was first suggested more than 40 years ago that the active properties of the Purkinje cell dendrite significantly influence the computation performed by this neuron, it has taken 40 years of combined modeling and experimental work to reach the beginning of an understanding about this relationship. Further, that emerging understanding suggests that much of our intuition over the last 40 years has been largely wrong. Changes in thinking about the physiological structure of the Purkinje cell dendrite has, in turn, driven, at least in our laboratory, a fundamental reconsideration regarding the function of the cerebellum as a whole (Bower, 1997a,b; Bower et al., 2012; for context for in the overall field see: Manto et al., 2012).

While general speculations on this subject can still be found in many experimental papers, the combination of realistic modeling and experimental studies described here has specifically revealed that Purkinje cell responses to granule cell-related excitatory and inhibitory synaptic inputs are quite different from the parallel fiber dominant, integrate and fire type cellular dynamics assumed by the most current theories of cerebellar function (Braitenberg, 1967; Marr, 1969; Albus, 1971; Pellionisz and Szentágothai, 1974; Medina and Mauk, 2000; Vetter et al., 2001; Heck and Sultan, 2002; Ito, 2006; Hong and Optican, 2008; Kitamura and Kano, 2012). In fact, while the 500+ references in the literature for the R-DB Model is very high compared to almost all models of its kind, in the last 20 years, there have actually been over 10,000 Purkinje cell experimental papers published, almost none of reference models of any kind. It is also completely standard, 40 years after Purkinje cell modeling started, for review articles on Purkinje cell function to make no mention what-so-ever of these modeling efforts or their results (e.g., from the last 2 years; Gallian and De Zeeuw, 2014; Grasselli and Hansel, 2014; Jörntell, 2014; Lewis and Raman, 2014; Voogd, 2014; Cerminara et al., 2015; Cheron et al., 2015; Dar, 2015; Louis, 2015; Tada et al., 2015). In fact, even review articles on subjects as central to the modeling as the active properties of the Purkinje cell dendrite can quite remarkably be published with hardly any mention of modeling results (Kitamura and Kano, 2012). Yet, many of the issues raised in those reviews, as well as the experimental papers they are based on raise issues that modelers have been investigating for years and many that have been resolved years ago.

How then are we to proceed in an organized way to understand function at any level, from the cerebellum itself down to the voltage dependent conductances in the Purkinje cell dendrite. This article is an example of how such study can proceed if based on realistic models shared by a community. Yet most published models are still designed to demonstrate a preexisting functional idea. In this regard, it is a remarkable fact that Pellionisz and Llinas first proposed more than 25 years ago a standard of “adequacy” for representing Purkinje cells (Pellionisz and Llinás, 1977). Yet most published models of Purkinje cells and certainly almost all published network models make no attempts what-so-ever to demonstrate that their Purkinje cells behave like actual Purkinje cells (Blum et al., 1993; Buonomano and Mauk, 1994; Yuen et al., 1995; Barto et al., 1999; Chauvet and Chauvet, 1999; Medina and Mauk, 2000; Spoelstra et al., 2000; Kistler and De Zeeuw, 2002; Brunel et al., 2004; Mauk and Ohyama, 2004; Yamazaki and Tanaka, 2007; Carrillo et al., 2008; Kulagina et al., 2008; de Gruijl et al., 2009; Abrams et al., 2010; Dean et al., 2010; Ohyama et al., 2010; Dean and Porrill, 2011; Li et al., 2012; Yamazaki and Nagao, 2012). It is entirely unclear what the value of a model is if the properties of its neurons, in this case a neuron with important active dendritic conductances, bears little resemblance to its actual physiological properties. Philosophers of science have long recognized the distinction between observation-based story telling and quantitative model-based analysis (Kuhn, 1962). In my view, models that misrepresent the actual physical properties of their neurons, including in this case usually neglecting the active properties of their dendrites, are essentially an extension of the story telling tradition. It is also worth noting that many of the models referenced above concern, perhaps, issues that many consider to be more directly related to cerebellar function, aging, learning, ataxia, effects of alcohol abuse, etc. These are clearly of interest to the cerebellar community, especially with the pressure for so-called translational science. In my view, a real understanding of these kinds of issues will absolutely depend on the continued construction and further elaboration of the level of realistic model described here, best done as part of a community. However, given the current state of the model, I see no reason why questions involving synaptic plasticity, pharmacological effects on specific ion channels, and even, possibly the kinds of aberrant behavior seen in Purkinje cells in some conditions of ataxia, can’t begin to be studied with a model of this type.

This in fact, is perhaps the most important reason that over the next 20 years it will be critical for the computational neuroscience community to adopt and build community models (Bower and Bower, 2013). If we are all simply working on our own disconnected individual models, we have little chance of establishing the kind of tested and accepted underlying quantitative framework that is likely essential for real scientific progress. By committing to the use of community models we also establish a common structure that can be presented to the larger neuroscience community, not as just another model, but as a model that has been built, tested, verified and accepted by multiple researchers. Why shouldn’t these models, then find their way into graduate training programs, or neuroscience textbooks? Why shouldn’t such a model be used as a standard against which other models are tested? As long as modelers fail to cooperate, they will likely continue to be largely ignored, not only be experimentalists, but also by their fellow modelers. It is only through the cooperative building and testing of models that an underlying quantitative infrastructure will begin to be constructed for neuroscience. In my view, the last 40 years demonstrates that it is only through that kind of infrastructure that we will ever understand complex phenomena, like, for example, the functional implications of active neuronal processes.

## Conflict of Interest Statement

The author declares that the research was conducted in the absence of any commercial or financial relationships that could be construed as a potential conflict of interest.
